# A Pilot mHealth Text Messaging Program Targeting Parents During the First 2000 Days: Nonrandomized Repeat Cross-Sectional Analysis to Evaluate Feasibility, Engagement, Acceptability, and Potential Effectiveness

**DOI:** 10.2196/83162

**Published:** 2026-06-15

**Authors:** Jacklyn Jackson, Nayerra Hudson, Tessa Delaney, Alison L Brown, Sienna Kavalec, Jessica Pinfold, Rebecca Sewter, Hannah McCormick, Rebecca Liackman, Christophe Lecathelinais, Lynda Davies, Luke Wolfenden, Daniel Groombridge, Belinda Tully, Sinead Redman, Tauri Smart, Nafiseh Ghafournia, Paul David Craven, Rachel Sutherland

**Affiliations:** 1 School of Medicine and Public Health, The University of Newcastle, University Drive, Callaghan, 2308, Australia, 61 0249246499; 2 Hunter Medical Research Institute, Newcastle, Australia; 3 Hunter New England Local Health District, New Lambton, Australia; 4 National Centre of Implementation Science, The University of Newcastle, Callaghan, Australia; 5 Armajun Health Service Aboriginal Corporation, Inverell, Australia; 6 School of Humanities, Creative Industries and Social Sciences, The University of Newcastle, Callaghan, Australia; 7 Western Sydney Local Health District, Paramatta, Australia

**Keywords:** digital health, mHealth, text message, first 2000 days, child health, feasibility, acceptability, cross-sectional

## Abstract

**Background:**

The first 2000 days can profoundly influence long-term health. Healthy Beginnings for Hunter New England Kids (HB4HNEKids) is an SMS text messaging program delivered alongside routine Child and Family Health Nursing (CFHN) care, which provides families with evidence-based, age- and stage-related preventive health information across the first 2000 days.

**Objective:**

This pilot study aimed to explore the feasibility, engagement, and acceptability of the HB4HNEKids program. It also aimed to explore the potential effectiveness of the program at 6 and/or 12 months post partum on outcomes including breastfeeding, child diet, child movement, and parental mental well-being.

**Methods:**

During the pilot phase (October 2021 to July 2024), project records were used to assess the number of families enrolled, number of SMS text messages sent (feasibility), and the number of opt outs (engagement). Repeat cross-sectional surveys were conducted at 5‐7 months post partum and again at 12‐14 months post partum using validated survey instruments. Using convenience sampling methods, survey participants consisted of birthing parents who had received HB4HNEKids and a concurrent nonrandomized comparison group that did not receive the program. Surveys assessed parental self-reported engagement with the messages, program acceptability, breastfeeding status, child diet, child movement, and parental mental well-being. Mixed linear regression analyses were conducted to calculate mean differences and odds ratios.

**Results:**

During the pilot phase, HB4HNEKids was delivered to 6243 families (73.4% of families contacted by CFHN). A total of 383 birthing parents completed the survey at 6 months (99/383, 26% receiving HB4HNEKids), and 283 completed the survey at 12 months (104/283, 37% receiving HB4HNEKids). Of the survey participants who received HB4HNEKids (n=200), between 76% and 83% reported that they always or very often read the SMS text messages, spending on average 5‐7 minutes engaged with the content. At both survey time points, more than 90% of participants receiving HB4HNEKids agreed that the program was acceptable. Child daily intake of vegetables was significantly higher in the HB4HNEKids group (adjusted mean difference 0.23, 95% CI 0.07-0.40; *P*=.006) than in the comparison group at 12 months. Parents receiving HB4HNEKids also reported significantly better mental well-being scores (*P*=.005). While HB4HNEKids participants reported breastfeeding rates 5 percentage points greater than comparison participants at 6 and 12 months, this result was not statistically significant. There were no statistically significant differences between HB4HNEKids, and comparison participant responses related to child movement behaviors.

**Conclusions:**

The HB4HNEKids SMS text messaging program is feasible to deliver at scale alongside routine CFHN care and is highly acceptable and engaging to parents. This pragmatic evaluation of the pilot, embedded into usual care, indicates potential effectiveness of the program for improving child vegetable intakes and parental mental well-being. Further evaluation of this program using robust methodology is needed to determine the effectiveness of this innovative mHealth program across the first 2000 days.

## Introduction

The first 2000 days of life represents a critical period between conception through to 5 years that significantly impacts on child’s physical, emotional, social, and cognitive development, laying the foundations for a child’s lifelong trajectories [1]. Breastfeeding practices, introduction to solid foods, development of movement behaviors, and optimal parental mental well-being can significantly influence child’s health and developmental outcomes [2-4]. The mental well-being of parents can influence the quality of care given to infants and is associated with a variety of health and developmental outcomes [5-7]. While longer breastfeeding duration, child’s consumption of fruits and vegetables, and increased physical activity are associated with enhanced cognitive development and reduced lifelong risk of chronic disease [2-4,8], current data suggest that these outcomes are below national and international targets [9-11].

In alignment with key international, national, and state-level health policies, Child and Family Health Nursing (CFHN) services in New South Wales (NSW), Australia, provide care and support to families following the birth of infants, with the goal of enhancing child and family health, development, and well-being during the first 2000 days [12,13]. The services offered by CFHN are primarily delivered face-to-face and align to scheduled health and developmental checks, providing lactation education, emotional support, vaccinations, preventive health advice, and screening and secondary referral pathways. However, due to various health service barriers (eg, staff capacity and geographic location), care focuses on supporting families during the first few months of the child’s life, with ongoing targeted services prioritized for those identified with additional needs. Furthermore, accessing face-to-face preventative health services can be challenging for parents, given caregiving responsibilities and work commitments [14,15]. Therefore, population-wide engagement with CFHN care significantly declines over the first 2000-day period, resulting in fragmented service utilization (NSW Ministry of Health, Health System Performance Report, internal data, 2025). This highlights the need for inclusive and accessible preventive health services to ensure that all families receive timely, trusted, and evidence-based support throughout the course of the first 2000 days.

Mobile health (mHealth) interventions represent a promising strategy to overcome some of the barriers to the traditional face-to-face support offered during the first 2000 days. Given that access to mobile phones and smartphones is relatively ubiquitous [16,17], mHealth interventions, including targeted SMS text messages delivered directly to parent or carer mobile phones, could improve the delivery of preventative care and health promotion during the first 2000 days [18]. This concept is supported by formative research conducted within the Hunter New England (HNE) region of NSW, Australia, which found that parents are highly accepting of receiving CFHN support via a variety of technology modalities (including websites, email, and SMS text messaging) on a variety of child– and family health–promoting topics (including healthy eating, introduction to solids, breastfeeding, sleep, and healthy growth) [19]. Systematic reviews also demonstrate that mHealth is effective in increasing multiple health behaviors including frequency of maternal contact with antenatal care, child immunization rates, and improving maternal and child health outcomes such as anxiety and depression, obesity prevention, and exclusive breastfeeding rates [20-25]. Additionally, a randomized controlled trial (RCT) conducted under robust research conditions demonstrates the efficacy of 1-way parent-targeted SMS text messaging interventions for improving infant feeding practices (ie, bottle use at bedtime) and reducing screen time [26]. This highlights the potential for 1-way SMS text message–based interventions during the postpartum period of the first 2000 days.

Despite the potential of mHealth interventions, few effective interventions have been embedded into health service delivery and implemented in real-world settings during the first 2000 days [27-29]. For mHealth interventions to effectively improve population health, they must be integrated into the settings, services, or health systems that reach the intended target population [29]. Given the limited translation of effective mHealth programs into usual health service delivery and building on the robust research evidence [18], there is a need to evaluate the feasibility, engagement, acceptability, and potential effectiveness of research innovations when embedded into routine care. The aim of this study was to pragmatically evaluate the feasibility, engagement, and acceptability of the pilot Healthy Beginnings for Hunter New England Kids (HB4HNEKids) program when embedded into routine CFHN service delivery. This pilot also explores the potential effectiveness of HB4HNEKids for improving child and parental health and well-being outcomes at 6 and/or 12 months post partum, including (1) breastfeeding, (2) child nutrition, (3) child movement, and (4) parental mental well-being.

## Methods

### Study Design

**Figure 1.**
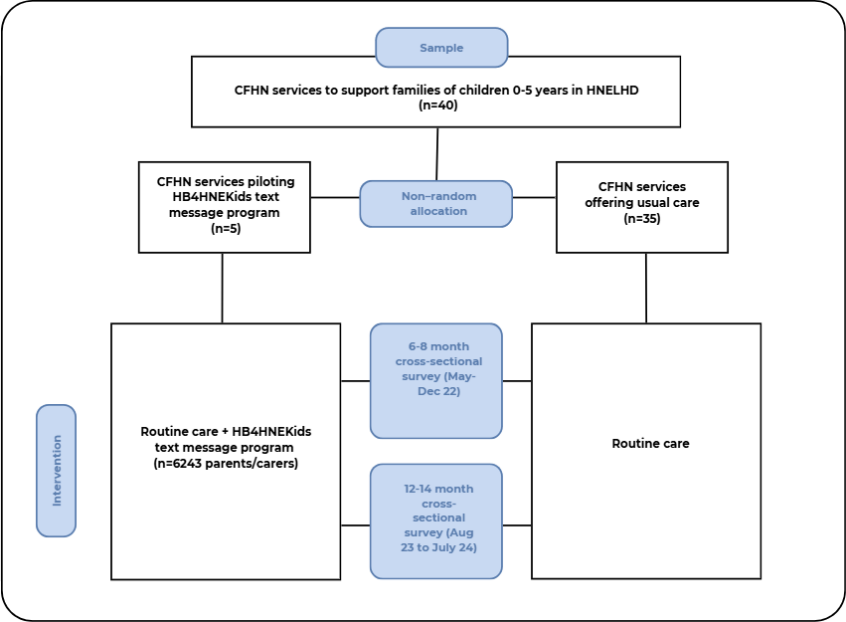
Flow of participants through the study. CFHN: Child and Family Health Nursing; HB4HNE: Healthy Beginnings for Hunter New England.

The feasibility, engagement, acceptability, and potential effectiveness of an SMS text messaging model of care delivered to families during the first 2000 days were evaluated as part of an observational study with a nonrandomized control arm. This study involved 2 cross-sectional surveys conducted with a convenience sample of birthing parents located in the Hunter New England Local Health District (HNELHD) of NSW, Australia. As the 1-way SMS text messaging program (HB4HNEKids) was initially launched in October 2021 to parents attending 5 pilot CFHN services across 3 diverse local government areas (Armidale, Cessnock, and Newcastle/Lake Macquarie), the cross-sectional surveys include a subsample of participants who had received HB4HNEKids and a concurrent non–randomized comparison group of parents who did not receive HB4HNEKids. The first cross-sectional survey was undertaken between May and December 2022 and included birthing parents 5‐7 months post partum. The second cross-sectional survey was conducted between August 2023 and July 2024 and included birthing parents 12‐14 months post partum (Figure 1).

This repeat cross-sectional study is reported in accordance with the STROBE (Strengthening the Reporting of Observational Studies in Epidemiology) guidelines for cross-sectional studies (Checklist 1).

### Ethical Considerations

Ethics approval for all maternal data was obtained from the Hunter New England Human Research Ethics Committee (16/11/16/4.07), Aboriginal Health and Medical Research Council (1236/16), and The University of Newcastle Human Research Ethics Committee (H-2017‐0032). This research was conducted in compliance with informed consent guidelines, and study participant informed consent was sought prior to completion of surveys and implied upon the completion of a survey. Participant data were collected and securely stored using the REDCap (Research Electronic Data Capture), compliant with health service privacy and confidentiality policies. Data were anonymized, deidentified, and stored so that only authorized individuals could access the data. This research did not offer participants compensation for participating in the SMS text messaging program (HB4HNEKids) or the surveys.

### Data Collection Procedures

#### Overview

Both the 6-month and 12-month surveys were developed using the REDCap (version 14.1.2; © 2025 Vanderbilt University) tool, hosted by HNELHD, which is a secure research electronic database commonly used in health service research [30,31]. Participants completed the survey either online or via computer-assisted telephone interview (CATI). Survey consent and responses were also stored in REDCap, providing a central survey database.

#### Eligibility for Participation in the Surveys

To evaluate the effectiveness of the pilot HB4HNEKids SMS text messaging program, a cross section of birthing parents who had accessed HNELHD maternity services was invited to participate in a survey at 2 time points. For the first survey, eligibility criteria for birthing parents were 26‐37 weeks (ie, approximately 6 months) post partum; those aged 18 years or older; had received antenatal care from any public antenatal service in HNE (providing antenatal care to approximately 70% of women across the district); had not had an unfortunate pregnancy-related outcome (ie, stillbirth); able to communicate in English; had previously provided consent to participate in a survey of antenatal care; and agreed to be contacted for future surveys. Eligibility for the second survey was the same as the first survey, except that birthing parents needed to be approximately 12 months post partum and have reached >34 weeks of gestation to be eligible to complete the survey.

#### Recruitment of Participants to Participate in the Survey

As per ethics approval, electronic medical records and previous antenatal survey data were used to identify the sample. Using this method, birthing parents who met eligibility criteria for survey participation were mailed printed information statements, providing an outline of the survey and a toll-free number to withdraw or decline participation.

##### Recruitment of Non-Aboriginal Participants to the Survey

Participants were sent information statements if their child met the eligibility criteria for participation. One week after information statements were mailed, birthing parents were contacted by telephone and invited to participate in the survey via a CATI. Birthing parents received up to 10 phone call attempts over a 2-week period to invite survey participation. As per ethics approval, verbal consent to participate in the surveys was sought from the birthing parent at the time of the interview. Birthing parents who declined to participate during the CATI were offered the opportunity to complete the survey online and were sent an individual survey link to their mobile number or email address. Before accessing the online survey, participants were reminded in the survey’s display screen that participation was voluntary and that it was possible to decline the survey at any point. Participant consent into the study and survey completion status (both via CATI and online) were saved into a central survey database, in REDCap, held by the research team.

##### Recruitment of Aboriginal Participants to the Survey

Following advice provided from local cultural consultation, birthing parents identifying as Aboriginal and/or Torres Strait Islander were sent an SMS text message 3 days after the mail out of the information statement. The SMS text message offered one of three options (1) to complete the survey via CATI, (2) to complete the survey online, or (3) decline participation. Participants could respond to the SMS text message to indicate their preferred method of contact. Those who opted to complete the survey online were sent an individual survey link to their mobile number which was active for 2 weeks. Those who opted to complete the survey via telephone or did not reply to the SMS text message within 5 days received a phone call from a female interviewer and were invited to participate in the study.

### The Text Messaging Program—Healthy Beginnings for Hunter New England Kids

#### Overview

HB4HNEKids is an mHealth SMS text messaging program providing parents or carers with age- and stage-relevant preventative health information. The program is an adapted version of an RCT that demonstrated impact on reducing screen time and bottle use at bedtime at 12 months [26]. The HB4HNEKids program was adapted to strengthen a range of behavioral outcomes, supporting parents and children to develop preventive health behaviors during the first 2000 days. Implementation focused on overcoming health system and parent barriers to accessing health services and was therefore designed to be delivered alongside routine CFHN care. HB4HNEKids aims to offer timely, age- and stage-appropriate health promotion information directly to parents’ and carers’ mobile phones including preventive health information such as breastfeeding, infant feeding, movement behaviors, and parental well-being. The program also offers reminders for scheduled child health checks and immunizations, early developmental screening, and information on available support services.

#### Program Adaptation Process

**Figure 2.**
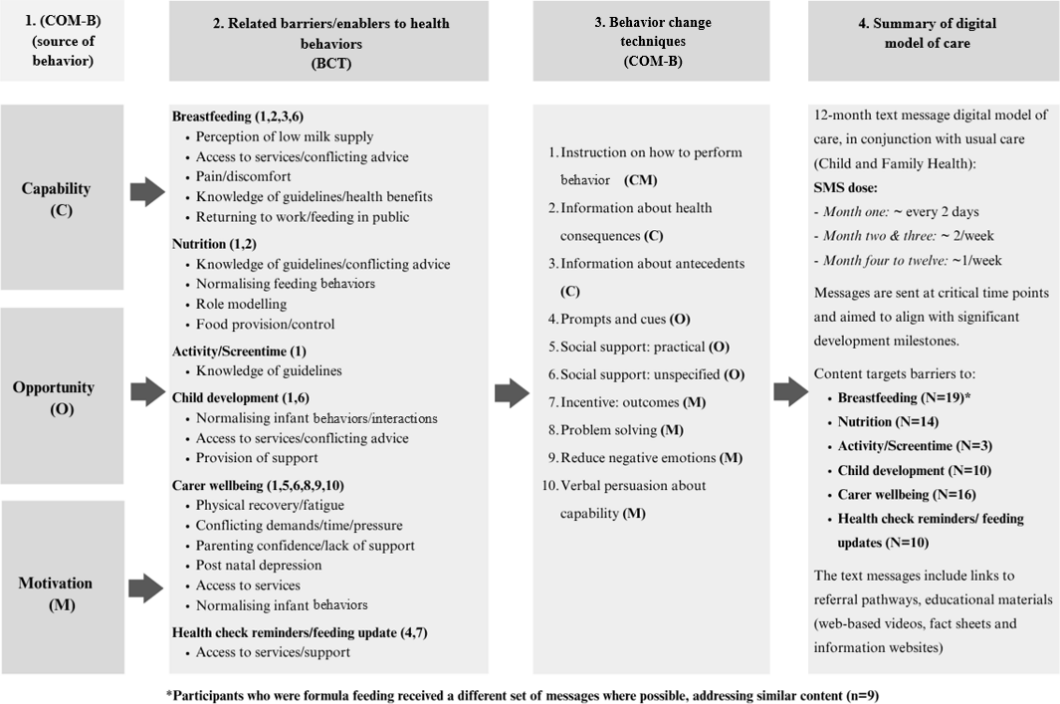
Healthy Beginnings for Hunter New England Kids logic model.

HB4HNEKids was adapted from a prior RCT [26] to strengthen the effectiveness of SMS text message support offered across the first 2000 days and enhance alignment to usual CFHN care delivery. The HB4HNEKids program focuses on 6 key health behaviors, aligned with specific age and stage developmental milestones and key policy frameworks targeting the first 2000 days [13,32,33]. The process of adaptation involved (1) identifying priority health behaviors from policy frameworks, strategic plans, and expert consultation; (2) review of the literature to identify determinants of priority health behaviors; (3) use of behavioral theory (eg, Behavior Change Wheel [34] and Theoretical Domains Framework [35]) to develop SMS text messages embedding behavior change techniques to address the identified determinants; and (4) co-design of SMS text messages collaboratively with HNELHD health practitioners including CFHN, allied health professionals, public health practitioners, Aboriginal health workers, and representatives from multicultural health to ensure that wording and intent aligned with usual care (Figure 2).

All eligible families were offered HB4HNEKids either prior to or during the face-to-face routine 1‐ to 4-week health check provided by CFHN services, as a digital program offered alongside usual care. The HB4HNEKids SMS text messages were short (often less than 160 characters) and included clickable links to evidenced-based “online” videos, fact sheets, websites, and relevant support services. Parents receiving the HB4HNEKids program were sent up to 44 SMS text messages in the first 6 months and a further 28 between 6 and 12 months to their nominated mobile number. Messages were sent 7 days a week, generally between the hours of 9 AM and 12 PM. While most messages were designed to be a 1-way SMS text message, the exception was the 2-way SMS text message sent to participants to confirm infant feeding status (on days 21, 97, and 150). Responses to feeding status were received in the messaging platform. Feeding status responses were stored in REDCap and monitored by a member of the research team each weekday. By collecting feeding status, the program content was tailored to better meet the individual needs of the participant (eg, families who were exclusively formula feeding did not receive content related to breastfeeding). Participants were sent a link on days 1, 51, and 296, which allowed participants to opt out and automatically unsubscribe from the program upon clicking. The opt out links remained valid or open allowing the participant to opt out of the program at any time.

#### Eligibility for Receipt of HB4HNEKids Program

All parents in HNELHD who had recently given birth are referred to CFHN services to receive ongoing care for their child until age 5 years. Parents due to receive a 1‐ to 4-week health check from 1 of the 5 participating CFHN services located in the HNELHD between October 2021 and July 2024 were eligible for the HB4HNEKids SMS text message program alongside their usual CFHN care. Parents needed to have access to a mobile phone to be eligible to receive the program.

#### Recruitment of Women to the HB4HNEKids Program

As per the existing health service procedures, CFHN services received a referral from public and private maternity services in the HNELHD following the birth of a baby and discharge of the parent from hospital [36]. CFHN service staff then contacted birthing parents in their local catchment area to offer a 1‐ to 4-week health check, which typically occurs within 3 business days of receipt of the CFHN referral. Staff from pilot CFHN services were encouraged to offer the HB4HNEKids program to all birthing parents within their catchment area. At the initial booking in contact, CFHN staff confirmed feeding status (breastfeeding, mixed feeding, and formula feeding), child’s name, mobile number, and “clinician offer of service” (yes, client refused, and unable to contact after multiple attempts), and recorded this in a REDCap [31]. If CFHN service staff were unable to determine infant feeding status during the initial call (ie, due to language barriers), they would locate infant feeding status via the discharge summary provided by maternity services and enter this into REDCap.

### Outcome Measures

#### Feasibility of Delivering HB4HNEKids via Routine CFH Care

During the relevant pilot time frame (October 2021 to July 2024), project record data (securely stored in REDCap) were used to assess feasibility of the program as follows: (1) number of families enrolled in the HB4HNEKids program, (2) proportion of families enrolled in the HB4HNEKids program relative to the number of families contacted by CFHN services for the 1‐ to 4-week appointment, (3) number of families who opted out of the HB4HNEKids program, and (4) number of messages sent as part of the HB4HNEKids program.

#### Engagement With HB4HNEKids Content

Within the 6-month and 12-month surveys, parents who had received HB4HNEKids were asked to report their engagement with the program SMS text message content. These items assessed how often participants read the SMS text messages and how often they clicked the website links within the SMS text messages, with response options including Always, Very often, Sometimes, Rarely, and Never. Participants were also asked to report average time spent (in minutes) engaged in the SMS text message content and links.

#### Acceptability of HB4HNEKids

To assess parent acceptability of the HB4HNEKids SMS text messaging program, measures of program acceptability were adapted from the psychometrically validated Acceptability of Intervention Measure by Weiner et al [37]. All survey items were assessed on a 5-point Likert scale (strongly disagree=1, disagree=2, neither agree nor disagree=3, agree=4, and strongly agree=5). Participants were coded as “agreeing” to statements, if they responded “strongly agree” or “agree” to any of the statements. Responses to Acceptability of Intervention Measure items were grouped according to (1) acceptability of the overall program, (2) acceptability of SMS text message timing, (3) acceptability of SMS text message content, and (4) acceptability of linked resources.

#### Potential Effectiveness

##### Breastfeeding Status, Introduction to Solids, and Breastfeeding Self-Efficacy at 6 and 12 Months

Infant feeding survey items were adapted from local, state, and national infant feeding surveys [38]. As per the Australian National Infant Feeding Survey, “current” breastfeeding status was reported if a parent reported “yes” to their “baby had received breastmilk since this time yesterday.” Breastfeeding duration was assessed in accordance with the Centers for Disease Control and Prevention definitions of breastfeeding duration [39], as parents who had ceased breastfeeding were asked to report the age (in months) at which breastfeeding ceased. Participants were also asked to report age (in months) the infant was first introduced to soft, semisolid or solid food, using survey items from the Australian National Infant Feeding Survey [38].

The Breastfeeding Self-Efficacy Scale—Short Form tool was used to assess maternal breastfeeding self-efficacy [40], which has consistently been demonstrated as a valid and reliable measure of breastfeeding self-efficacy globally [41]. The Breastfeeding Self-Efficacy Scale—Short Form is a 14-item self-report instrument derived from the 33-item Breastfeeding Self-Efficacy Scale tool measuring breastfeeding confidence. All items are preceded by the phrase “I think I can always” and rated on a 5-point Likert scale, ranging from 1 (not at all confident) to 5 (always confident). As such, total score ranges from 14 to 70, with a higher score indicating greater levels of breastfeeding self-efficacy. The items were assessed within both the 6-month and 12-month surveys.

##### Child Nutrition at 12 Months

The Short Food Survey (SFS) developed by Hendrie et al [42] is a 38-item tool that captures parent-reported information on children’s food group intakes and food choices and has been shown to produce a reliable estimate of compliance with dietary guidelines [42]. Four survey items from the SFS were used to assess child intake of fruit and vegetables at 12 months. Fruit intake was assessed by asking parents “How many serves of fruit does your child usually eat,” including a prompt describing what a serve of fruit is (eg, 1 serve is equal to 2 small pieces of fruit, eg, apricots and plums; 1 medium apple, banana, or mandarin; or 1 cup of diced pieces, eg, diced peaches or diced fruit salad). To assess child intake of vegetables, responses to 3 survey items from the SFS were combined [42], including “How many servings of starchy vegetables (not including hot chips) does your child usually eat?” (prompt: starchy vegetables include potatoes and sweet potatoes; 1 serve is equal to ½ a cooked potato); “How many servings of salad vegetables does your child usually eat?” (prompt: salad vegetables include vegetables such as tomato, capsicum, cucumber, lettuce, and celery. A serve is equal to 1 cup of salad vegetables); and “How many servings of cooked vegetables does your child usually eat?” (prompt: a serve is equal to ½ cup of cooked vegetables).

The Children’s Dietary Questionnaire [43] is a survey tool used to reliability assesses child intake patterns in either the previous week or 24 hours. We used survey items from the Children’s Dietary Questionnaire to measure the mean weekly frequency of discretionary food consumption, captured by combining responses related to the frequency of child’s intake of soft drinks (including cordial or sports drinks), takeaway foods (meals or snacks), chips (eg, baked potato gems/chips, hash browns, hot chops or French fries, wedges, or fried potatoes), savory snacks (eg, potato crisps, pretzels, or plain or flavored crackers), sweet snacks (eg, sweet biscuits, cakes, buns, muffins, and doughnuts), savory pastries, snack bars (eg, muesli bars or cereal bars), chocolate or lollies, and ice cream or ice-blocks. Items assessing child nutrition were included only within the 12-month survey.

##### Child Movement Behaviors at 12 Months

The Movement Behavior Questionnaire—Baby (MBQ-B) is a validated 6-item tool designed to be administered to parents of children younger than 18 months [44]. The MBQ-B assess frequency and duration of tummy time, time spent in active play or outdoor play, watching television, and using mobile digital devices. Items from the MBQ-B were used to assess child movement, restrained time, and screen time.

For active play, parents were asked “Thinking about the past week, on a typical day, how much time in total did you do some active play with your child? Active play could be crawling on the floor with your child, rolling around the floor with your child, playing at the park, dancing with your child, chasing your child.*”* The response options ranged from 0 minutes per day to more than 2 hours per day. Given Australian 24-hour movement, guidelines recommend that toddlers (aged 1‐2 years) should achieve at least 3 hours of various physical activities each day, including energetic play, and parents reporting “more than 2 hours per day” of active play were coded as having children meeting physical activity guidelines [45]. To explore restrained time, parents were asked to report “On a typical day, how many times did you place your child in a baby carrier or sling, car seat or capsule, stroller or pram, highchair, bouncer, jolly jumper or play pen?,” and “When thinking about each occasion that your child was in one of those devices, how long were they usually in it?.” Given that the infant movement guidelines recommend not restraining an infant for more than 1 hour at a time [45], response options of “less than 15 minutes per day,” “between 15 and 30 minutes per day,” “between 30-35 minutes per day,” and “between 45 to 60 minutes per day” were combined to indicate the proportion of parents compliant with this recommendation. Screen time behaviors were explored by asking participants 2 surveys items: “Thinking about the past week, on a typical day, how much time did your child spend watching television programs, videos/internet clips or movies on a television, computer or portable/mobile device such as iPad, tablet or smartphone?*”* (ie, passive screen time) and “Thinking about the past week, on a typical day, how much time did your child spend playing games, looking at photos, or video chatting (eg, FaceTime, Zoom, Skype) on a screen-based device such as a computer or laptop, video games console, iPad, tablet, or smartphone?” (ie, interactive screen time). Response options ranged between “0 minutes per day” and “more than 2 hours per day.” Given Australian 24-hour movement guidelines recommend no screen time for children younger than 2 years [45], if a parent responded “0 minutes per day” to screen time items, they were coded as “not offering any screen time per day” indicating the proportion of children meeting screen time guidelines. These items were assessed only within the 12-month survey.

##### Parental Mental Well-Being at 12 Months

The Short Warwick Edinburgh Mental Wellbeing Scale (SWEMWBS) was used to capture parental mental well-being [46]. The SWEMWBS was funded by the Scottish Executive National Program for improving mental health and well-being, commissioned by NHS Health Scotland, developed by the University of Warwick and the University of Edinburgh, and is jointly owned by NHS Health Scotland, the University of Warwick, and the University of Edinburgh [47]. The SWEMWBS is a 7-item tool that has been positively worded to measure participant thoughts and feelings related to functioning over the past 2 weeks. The SWEMWBS is scored by summing the scores for each of the 7 items (scored 1 “none of the time” to 5 “all of the time”) and then transformed into metric scores using the SWEMWBS conversion table [46]. Scores can range from 7 to 35, with higher scores indicating higher positive mental well-being, and has been shown to be a valid and reliable measure of mental well-being among caregivers [48]. The SWEMWBS was assessed within the 12-month survey only.

### Participant Demographics and Pregnancy Characteristics

Within the survey, birthing parents were asked questions related to Aboriginal and/or Torres Strait Islander origin, country of birth, residential postcode, current employment status (full-time, part-time, casual, paid or unpaid maternity leave, unemployed, home duties, studies, retired, full-time carers, and unable to work due to health problems), timing of their return to work after birth (in months), and the child’s date of birth. The survey items are based on those used in previous surveys with postpartum women [49]. Participant education status, first or subsequent pregnancy status, and maternal age data had been collected via previous surveys or medical record data. Participant residential postcode was used to determine geographical remoteness (“major cities” or “regional or remote”) using the Accessibility/Remoteness Index of Australia [50]. Postcode was also used to determine socioeconomic areas (“most disadvantaged” or “least disadvantaged”) using 2016 socioeconomic indexes for areas [51].

### Data Analysis

Data were analyzed by an independent statistician using SAS statistical software (version 9.3, SAS Institute). Descriptive statistics were used to describe (1) characteristics of the samples, (2) feasibility outcomes, (3) program engagement outcomes, and (4) acceptability outcomes. Chi-square test or *t* test was used to compare the characteristics of participants between those who had received HB4HNEKids and those who had not (usual care control).

Sample size calculations were not conducted for this pilot feasibility study [52]. However, to explore the potential impact of the program on child breastfeeding, nutrition, and movement behaviors, as well as parental well-being outcomes (effectiveness), separate logistic regression analyses were conducted to examine differences between survey respondents who had received the HB4HNEKids program and those who had not (usual care control). Unadjusted and adjusted analyses were conducted at both time points. Analyses were adjusted for a range of key participant characteristics including parent age, Aboriginal and Torres Strait Islander status, highest level of education, socioeconomic status, and level of remoteness. Participants with missing data were excluded from analyses. Dichotomous outcomes were presented as odds ratios and 95% CIs, and continuous outcomes were presented as mean differences (MDs) and 95% CIs. A *P* value of <.05 was considered statistically significant.

## Results

### Feasibility of Delivering HB4HNEKids via Routine CFHN Care

During the 2.5-year pilot period (October 2021 to July 2024) of the HB4HNEKids program a total of 8501 families across the 5 CFHN services were eligible to receive the program. A total of 6243 families commenced the program (6243/8501, 73.4%), from which 430 families later withdrew, giving a 6.9% (430/6243) dropout rate. A total of 377,969 SMS text messages were sent during the pilot period (average of 61 messages per family).

### Characteristics of Cross-Sectional Survey Participants

Of the 780 birthing parents approached, a total of 383 (49% response rate) eligible participants completed the 6-month survey, whereby 26% (99) of survey participants had received the HB4HNEKids program. Of the 626 birthing parents approached, a total of 283 (45% response rate) participants completed the 12-month survey, from which 37% (104) survey participants had received the HB4HNEKids program. The 2 cross-sectional samples were similar in terms of birthing parent’s age, education status, socioeconomic status, and remoteness of geographical location (Table 1). The 12-month sample included a higher proportion of participants identifying as Aboriginal and/or Torres Strait Islander (66/283, 23%), compared with the 6-month sample (43/383, 11%). By 12 months the proportion of participants on parental leave had significantly dropped, with 8% (22/283) of participants on parental leave, compared with 45% (173/383) at 6 months. Correspondingly, more participants engaged in employment at 12 months (188/283, 66% vs 114/383, 30%, respectively).

**Table 1.** Characteristics of birthing parents who participated in the 6- and 12-month survey by HB4HNEKids participation^a^.

Characteristics	6-Month survey participants			12-Month survey participants		
	HB4HNEKids^b^	Usual care	Overall sample	HB4HNEKids	Usual care	Overall sample
Parents, n (%)	99 (26)	284 (74)	383 (100)	104 (37)	179 (63)	283 (100)
Age of parent (years), mean (SD)	30.6 (4.9)	30.6 (4.5)	30.6 (4.6)	31.6 (4.0)	30.9 (5.3)	31.2 (4.9)
Age of infant (months), n (%)						
5	2 (2)	5 (2)	7 (2)	—^c^	—^c^	—^c^
6	97 (98)	279 (98)	376 (98)	—^c^	—^c^	—^c^
12	—^c^	—^c^	—^c^	69 (66)	128 (71)	197 (70)
13	—^c^	—^c^	—^c^	30 (29)	41 (23)	71 (25)
14	—^c^	—^c^	—^c^	5 (5)	10 (6)	15 (5)
Aboriginal and/or Torres Strait Islander, n (%)						
Yes	8 (8)	35 (12)	43 (11)	10 (10)	56 (31)	66 (23)^d^
No	91 (92)	249 (88)	340 (89)	94 (90)	123 (69)	217 (77)^d^
Highest level of education, n (%)						
High school or less	21 (21)	79 (28)	100 (26)	18 (18)	57 (32)	75 (27)^d^
Tertiary	78 (79)	205 (72)	283 (74)	84 (82)	121 (68)	205 (73)^d^
Employment status, n (%)						
Employed	25 (25)	89 (31)	114 (30)	75 (72)	113 (63)	188 (66)
Maternity leave (paid or unpaid)	49 (50)	124 (44)	173 (45)	8 (8)	14 (8)	22 (8)
Unemployed	25 (25)	71 (25)	96 (25)	21 (20)	52 (29)	73 (26)
Socioeconomic status, n (%)						
Most disadvantaged	49 (49)	191 (68)	240 (63)^d^	42 (41)	119 (67)	161 (58)^d^
Least disadvantaged	50 (51)	90 (32)	140 (37)^d^	61 (59)	58 (33)	119 (42)^d^
Level of remoteness, n (%)						
Major city	78 (79)	156 (56)	234 (62)^d^	77 (75)	108 (61)	185 (66)^d^
Regional or remote	21 (21)	125 (44)	146 (38)^d^	26 (25)	69 (39)	95 (34)^d^

a Total 6-month sample (n=383): incomplete responses or missing data for socioeconomic status (n=380) and level of remoteness (n=380). Total 12-month sample (n=283): incomplete responses or missing data for highest level of education (n=280), socioeconomic status (n=280), and level of remoteness (n=280).

b HB4HNEKids: Healthy Beginnings for Hunter New England Kids.

c Not available.

d Statistically significant (*P*<.05) difference for the demographic characteristic between survey participants who had received HB4HNEKids and those who had not (usual care control group).

Participants who had received HB4HNEKids at the 6-month survey had a higher proportion from “least disadvantaged” areas and higher proportion residing in major cities than usual care control participants. Similar differences between groups were found at 12 months. Additionally, HB4HNEKids participants had a lower proportion of participants identifying as Aboriginal and/or Torres Strait Islander and a higher proportion of participants who had completed tertiary education.

### Engagement With HB4HNEKids Text Message Content at 6 and 12 Months

Of the survey participants receiving the HB4HNEKids program (n=200), 76% (73/96) and 83% (86/104) of participants from the 6-month and 12-month survey, respectively, reported that they always or very often read the HB4HNEKids SMS text messages. Participants from the 6-month survey reported spending an average of 7.43 minutes engaged in the SMS text message content of HB4HNEKids, while participants from the 12-month survey reported an average of 5.83 minutes engaged in the content. In addition, 43% (41/96) of participants from the 6-month survey and 31% (32/104) of participants from the 12-month survey reported that they always or very often clicked on the website links provided in the messages (Table 2).

**Table 2.** Self-reported engagement with HB4HNEKids^a^ SMS text message content and links.

	6-Month survey participants (n=96^b^)	12-Month survey participants (n=104)
Parent always or very often		
Read the SMS text messages, n (%)	73 (76)	86 (83)
Clicked the website links within the SMS text messages, n (%)	41 (43)	32 (31)
Minutes on average spent engaged in the SMS text message content and links, mean (SD)	7.43 (7.91)	5.83 (5.52)

a HB4HNEKids: Healthy Beginnings for Hunter New England Kids.

b Response data missing for 3 survey participants at 6 months, as those who reported opting out of the HB4HNEKids program were not required to provide data on SMS text message engagement.

### Acceptability of HB4HNEKids

At 6 months and 12 months, most parents reported that the program was acceptable (Table 3), with more than 90% of parents agreeing or strongly agreeing with statements related to the overall acceptability of the program, timing of the messages, and message content. More than 80% of parents found the embedded website links acceptable.

**Table 3.** Acceptability of the HB4HNEKids^a^ SMS text messaging program.

Aim and item	6-Month survey (agree or strongly agree; n=96^b^), %	12-Month survey (agree or strongly agree; n=104), %
The overall program		
Overall, I liked the HB4HNEKids program.	93	95
Overall, the HB4HNEKids program meets my approval.	96	94
Overall, the HB4HNEKids program was appealing to me.	—^c^	90
Overall, I was satisfied with the HB4HNEKids program.	97	93
I would recommend the HB4HNEKids program to other parents/carers.	94	93
SMS text message timing		
I was happy with the timing of the delivery of the content of text messages based on [child] age and development.	97	99
SMS text message content		
I found the immunization and growth check reminder text messages acceptable.	95	91
I found the information and advice on breastfeeding/formula feeding acceptable.	95	90
I found the information and advice on introduction to solids acceptable.	95	90
Linked resources		
I like the website links.	84	86

a HB4HNEKids: Healthy Beginnings for Hunter New England Kids.

b Response data missing for 3 survey participants at 6 months, as those who reported opting out of the HB4HNEKids program were not required to provide data on program acceptability.

c Not available.

### Potential Effectiveness

#### Breastfeeding Status, Breastfeeding Duration, Introduction to Solids, and Breastfeeding Self-Efficacy

The proportion of parents currently breastfeeding was not significantly different based on whether the parent had participated in the HB4HNEKids program or not at both time points (6 months and 12 months). However, at both time points, HB4HNEKids participants reported a 5% higher point prevalence of breastfeeding than the comparison group participants (6 months: 66% vs 61%; 12 months: 46% vs 41%). There was no difference in breastfeeding duration for HB4HNEKids participants compared with comparison participants. Similarly, there were no statistically significant differences between HB4HNEKids and comparison participants at both time points for mean age of solids introduction and breastfeeding self-efficacy scores (Tables4  5).

**Table 4.** Breastfeeding and infant feeding outcomes measured at 6 months by HB4HNEKids^a^ participation.

	HB4HNEKids	Usual care	Unadjusted estimate (95% CI), *P* value	Adjusted estimate^b^ (95% CI), *P* value
Proportion currently breastfeeding, n (%)	61 (66)	166 (61)	OR^c^ 1.23 (0.75 to 2.03), .41	OR 1.07 (0.62 to 1.83), .81
Breastfeeding duration in months, mean (SD)	3.80 (2.19)	3.86 (2.15)	MD^d^ –0.06 (–0.57 to 0.46), .83	MD –0.17 (–0.69 to 0.35), .53
Age in months child was introduced to first solid foods, mean (SD)	5.36 (0.80)	5.32 (0.87)	MD 0.04 (–0.16 to 0.24), .70	MD 0.06 (–0.14 to 0.27), .55
Breastfeeding self-efficacy score, mean (SD)	57.38 (12.72)	55.96 (14.36)	MD 1.41 (–1.90 to 4.73), .40	MD 1.30 (–2.12 to 4.73), .45

a HB4HNEKids: Healthy Beginnings for Hunter New England Kids.

b Adjusted analysis was adjusted for participant socioeconomic status, level of remoteness, level of education, Aboriginal and Torres Strait Islander status, and age.

c OR: odds ratio.

d MD: mean difference.

**Table 5.** Breastfeeding and infant feeding outcomes measured at 12 months by HB4HNEKids^a^ participation.

	HB4HNEKids	Usual care	Unadjusted estimate (95% CI), *P* value	Adjusted estimate^b^ (95% CI), *P* value
Proportion currently breastfeeding, n (%)	46 (46)	68 (41)	OR^c^ 1.23 (0.74 to 2.03), .43	OR 1.07 (0.62 to 1.87), .80
Breastfeeding duration in months, mean (SD)	3.07 (2.59)	3.24 (2.45)	MD^d^ –0.18 (–0.80 to 0.45), .58	MD –0.37 (–1.03 to 0.29), .27
Age in months child was introduced to first solid foods, mean (SD)	5.59 (1.02)	5.52 (1.02)	MD 0.06 (–0.18 to 0.31), .61	MD 0.02 (–0.24 to 0.29), .87
Breastfeeding self-efficacy score, mean (SD)	55.48 (15.77)	55.89 (15.34)	MD –0.40 (–4.30 to 3.50), .84	MD –0.73 (–4.82 to 3.37), .73

a HB4HNEKids: Healthy Beginnings for Hunter New England Kids.

b Adjusted analysis was adjusted for participant socioeconomic status, level of remoteness, level of education, Aboriginal and Torres Strait Islander status and age.

c OR: odds ratio.

d MD: mean difference.

#### Child Nutrition at 12 Months

Child daily intake of vegetable serves was significantly higher in the HB4HNEKids group in both the unadjusted (MD 0.21, 95% CI 0.05-0.36; *P*=.009) and adjusted analyses (MD 0.23, 95% CI 0.07-0.40; *P*=.006) than in the comparison group. Child daily intake of fruit serves was significantly higher in the HB4HNEKids group than in the comparison group participants in the unadjusted analysis (MD 0.28, 95% CI 0.05-0.50; *P*=.02); however, this result did not remain statistically significant in the adjusted analyses (MD 0.20, 95% CI –0.04 to 0.45; *P*=.10). There was no difference in the frequency of child discretionary food intake per week between the groups (Table 6).

**Table 6.** Child nutrition, child movement, and parental mental well-being outcomes measured at 12 months by HB4HNEKids^a^ participation.

	HB4HNEKids	Usual care	Unadjusted estimate (95% CI), *P* value	Adjusted estimate^b^ (95% CI), *P* value
Daily intake of fruit serves, mean (SD)	2.07 (1.01)	1.79 (0.88)	MD^c^ 0.28 (0.05 to 0.50), .02	MD 0.20 (–0.04 to 0.45), .10
Daily intake of vegetable serves, mean (SD)	0.74 (0.70)	0.53 (0.59)	MD 0.21 (0.05 to 0.36), .01	MD 0.23 (0.07 to 0.40), .006
Frequency of discretionary food intake per week, mean (SD)	0.99 (0.93)	1.17 (1.03)	MD –0.18 (–0.42 to 0.06), .15	MD –0.02 (–0.27 to 0.23), .88
Proportion meeting physical activity guidelines, n (%)	88 (85)	139 (78)	OR^d^ 1.69 (0.88 to 3.25), .12	OR 1.71 (0.84 to 3.48), .14
Proportion meeting restrained time guidelines, n (%)	70 (72)	131 (82)	OR 0.57 (0.31 to 1.05), .07	OR 0.62 (0.33 to 1.18), .15
Proportion meeting screen time guidelines, n (%)	24 (24)	48 (27)	OR 0.86 (0.49 to 1.51), .61	OR 0.63 (0.34 to 1.16), .14
Parental mental well-being scores, mean (SD)	26.15 (3.37)	24.22 (4.43)	MD 1.93 (0.94 to 2.92), <.001	MD 1.52 (0.47 to 2.58), .005

a HB4HNEKids: Healthy Beginnings for Hunter New England Kids.

b Adjusted analysis was adjusted for participant socioeconomic status, level of remoteness, level of education, Aboriginal and Torres Strait Islander status, and age.

c MD: mean difference.

d OR: odds ratio.

#### Child Movement at 12 Months

Table 6 includes unadjusted and adjusted analyses exploring the odds of participants meeting physical activity guidelines, restrained time guidelines, and screen time guidelines. There were no statistically significant differences between the proportion of HB4HNEKids participant and comparison participant responses related to meeting child movement guidelines (Table 6).

#### Parental Mental Well-Being at 12 Months

At 12 months, parents who had received the HB4HNEKids program reported a mean SWEMWBS score of 26.15, and comparison participants reported a mean SWEMWBS score of 24.2. Unadjusted and adjusted analyses indicate that parents who had received the HB4HNEKids program reported statistically significantly better mental well-being scores (unadjusted MD 1.93, 95% CI 0.94- 2.92, *P*<.001; adjusted MD 1.52, 95% CI 0.47-2.58, *P*=.005) than the comparison participants (Table 6).

## Discussion

### Principal Results

This study sought to evaluate the feasibility, engagement, and acceptability of the pilot HB4HNEKids program and explore its potential effectiveness on a range of child and parental health and well-being outcomes including breastfeeding, child nutrition, child movement, and parental mental well-being. Findings indicated that the program was feasible to deliver to families in HNELHD via CFHN services and is met with high acceptability and strong engagement from parents. Furthermore, the evaluation found that the program was likely effective in improving child vegetable intakes and parental mental well-being at 12-months. These findings provide support for further development and improvement of the program with formal testing as part of a larger RCT.

### Comparison With Prior Work

This pragmatic evaluation demonstrates that the 1‐ to 4-week CFHN service visit (attended by >80% of families in HNELHD) is a feasible way of enrolling new families into a mHealth program at scale. There remains scope to further optimize the reach of HB4HNEKids, given just 73.4% (6243/8501) of eligible families coming through the pilot CFHN services were enrolled into the program. However, the initial reach of the HB4HNEKids program appears relatively high in contrast with other digital health interventions (DHIs), with systematic reviews of DHIs reporting a median reach of just 21.8%‐33.6% [53]. Engagement is another critical factor in determining the success of DHIs, with systematic review evidence suggesting that engagement is associated with a variety of improved health behaviors including fruit and vegetable intake, weight status, smoking, and physical activity [54-56]. Our results indicate good participant engagement with the HB4HNEKids program, with most HB4HNEKids participants reporting that they “always” or “very often” read the SMS text messages, spending on average of 6 minutes interacting with each message. While a systematic review by Grady et al [57] found that SMS text messages are a key DHI design feature associated with engagement, the extensive co-design process undertaken in the design of the HB4HNEKids program may also be driving strong engagement rates [58]. This combined with low opt-out rates and high parent-reported acceptability, the HB4HNEKids program holds significant promise for reaching and engaging parents in health promotion content during the first 2000 days.

Vegetable intake is a key indicator of a healthy diet providing an important source of essential vitamins and minerals [59]; however, only 18.5% of Australian children aged 2‐3 years meet the recommended intake for vegetables, dropping to just 3.8% in children aged 4‐8 years [60]. Although data collected from parents within the HNE region of NSW, Australia, indicate that children aged 12 months are not meeting the recommended serves of vegetables each day (2‐3 serves), the evaluation indicates that children from families who were receiving HB4HNEKids were having 0.23 of a serve more vegetables per day than those from families in the usual care group [61] and represents an effect size consistent with other interventions that have demonstrated efficacy at increasing children’s vegetable intake in home and community settings [62]. Although this effect size is modest in absolute terms, this increase aligns with the magnitude of change commonly observed in early life dietary interventions and reflects a behavioral shift that is difficult to achieve during the introduction-to-solids period [63,64]. Small increments in vegetable intake at this developmental stage may nonetheless carry clinical relevance, as early exposure contributes to flavor acceptance, supports the establishment of healthier dietary trajectories, and enhances intake of key micronutrients such as folate, vitamin A precursors, and potassium [59]. Evidence indicates that even limited increases in vegetable consumption can reduce the risk of micronutrient inadequacy [65] and are prospectively associated with healthier dietary patterns linked to lower noncommunicable disease risk in adulthood [66]. Therefore, future research to assess the sustainment of the program’s effects over time is warranted to better quantify the child- and population-level benefit of the HB4HNEKids program.

Like other trials such as the original Health Beginnings RCT [26], our evaluation did not indicate a statistically significant difference in current breastfeeding rates between the HB4HNEKids and usual care participants. These findings are inconsistent with those of a recent systematic review of SMS text message–based interventions, with meta-analyses showing that they were effective at improving exclusive breastfeeding rates particularly when they engage families in the prenatal period [25]. However, given our pilot study was not powered for this outcome and still observed a 5-point prevalence difference in current breastfeeding rates (favoring HB4HNEKids participants) at 6 months and 12 months, this result is nonetheless highly encouraging. Even so, further development and optimization of the breastfeeding content offered within the program, or expanding the program to engage families earlier (ie, antenatally), could be of value.

In contrast with the original Healthy Beginnings RCT that found that families receiving SMS text message support (combined with accompanying printed resources) were significantly more likely to “never have screen time” than the control (adjusted odds ratio 1.28, 95% CI 1.08‐1.52; *P*<.05) [26], our evaluation did not indicate any differences between HB4HNEKids and usual care participants screen time behaviors or any other movement behaviors at 12 months. While the SMS text message content of the HB4HNEKids program related to child screen time and movement behavior during the first 12 months mirrored that of the original Healthy Beginnings SMS text message content and focused on increasing parental knowledge of child movement and screen time guidelines (eg, 1 message on movement guidelines, 1 message on restrained time guidelines, and 1 message on screen time recommendations), the omission of the booklet content from HB4HNEKids could be driving this difference. However, given that formative research suggests that parents would prefer to be engaged in content focused on the benefits of certain movement and screen time behaviors, rather than the risks [67], there remains opportunity to strengthen parent-directed SMS text message content to better address known barriers and facilitators to child screen time and movement behaviors during the first 12 months.

The perinatal period demands significant emotional, psychological, social, and physical adaptation from parents and represents a period of change that can have implications on parental well-being and mental health outcomes. Parental mental health and well-being are of key importance during this early life stage, as it can influence parent-child relationships, the quality of care provided, and in turn the child’s life trajectory [68-70]. Outside of the standardized mental health screening conducted by primary care services, there is limited support embedded within health services to generally support parental well-being [71,72]. Thus, the improved mental well-being scores at 12 months among those receiving the program compared with those who did not (MD 1.51, 95% CI 0.45-2.57; *P*=.005) are important.

### Limitations

The results of this evaluation must be considered within the context of our study limitations. These include the use of our convenience sampling methods, which included only a small subsample of participants who had received the HB4HNEKids program, thus not providing data that are representative of the whole HNE region. Furthermore, given our response rates of approximately 45%‐49%, our findings may be subject to nonresponse bias. Given the cross-sectional nature of our evaluation, and that HB4HNEKids and comparison participants were not randomly allocated, we cannot be certain that the differences in child vegetable intakes and parental mental well-being outcomes are explicitly due to the SMS text messaging program [73]. Furthermore, most of the outcomes assessed within this evaluation were self-reported by participants and may be influenced by self-report, social desirability, or recall biases. However, where possible, validated survey tools were used. This pilot study was not powered to detect statistically significant differences between groups for many of the outcomes assessed, including proportion of currently breastfeeding and proportion of children meeting physical activity guidelines. The HB4HNEKids program is currently operating as a complimentary mHealth program alongside an existing routinely offered health service. The insight offered through this evaluation of a real-world health service intervention (while acknowledging the limitations stated) would be strengthened with the opportunity to evaluate the program using more robust methodologies and a larger sample size.

### Conclusions

This evaluation of the pilot HB4HNEKids SMS text messaging program indicates that the program is feasible to deliver at scale, alongside routine CFHN care and is highly acceptable and engaging to parents. Our findings indicate possible efficacy of the program for improving child vegetable intake and parental mental well-being at 12 months. However, further evaluation of the program within larger representative samples using robust methodology is needed to determine the true impact of this innovative program.

## Supplementary material

10.2196/83162 Checklist 1 STROBE checklist for cross-sectional studies.

## Abbreviations

CATI: computer-assisted telephone interview
CFHN: Child and Family Health Nursing
DHI: digital health intervention
HB4HNEKids: Healthy Beginnings for Hunter New England Kids
HNE: Hunter New England
HNELHD: Hunter New England Local Health District
MBQ-B: Movement Behavior Questionnaire—Baby
MD: mean difference
mHealth: mobile health
NSW: New South Wales
RCT: randomized controlled trial
REDCap: Research Electronic Data Capture
SFS: Short Food Survey
STROBE: Strengthening the Reporting of Observational Studies in Epidemiology
SWEMWBS: Short Warwick Edinburgh Mental Wellbeing Scale
