# Supplementary material for: A Pilot mHealth Text Messaging Program Targeting Parents During the First 2000 Days: Nonrandomized Repeat Cross-Sectional Analysis to Evaluate Feasibility, Engagement, Acceptability, and Potential Effectiveness
Source: JMIR Mhealth Uhealth. 2026 Jun 15;14:e83162. doi: 10.2196/83162 (PMC13270169; doi:10.2196/83162)
Supplement: Checklist 1 [file mhealth-v14-e83162-s001.pdf]

STROBE Statement—checklist of items that should be included in reports of observational studies

	Item No.	Recommendation	Page No.	Relevant text from manuscript
**Title and abstract**	1	(*a*) Indicate the study's design with a commonly used term in the title or the abstract	1	**Title**
		(*b*) Provide in the abstract an informative and balanced summary of what was done and what was found	1	**Abstract –** Methods & Results
**Introduction**				
Background/rationale	2	Explain the scientific background and rationale for the investigation being reported	2-3	**Introduction** – paragraphs 1, 3 & 4
Objectives	3	State specific objectives, including any prespecified hypotheses	3	**Introduction** – paragraph 4
**Methods**				
Study design	4	Present key elements of study design early in the paper	4	**Methods –** Study Design
Setting	5	Describe the setting, locations, and relevant dates, including periods of recruitment, exposure, follow-up, and data collection	4	**Methods –** Study Design
Participants	6	(*a*) *Cohort study*—Give the eligibility criteria, and the sources and methods of selection of participants. Describe methods of follow-up<br>*Case-control study*—Give the eligibility criteria, and the sources and methods of case ascertainment and control selection. Give the rationale for the choice of cases and controls<br>*Cross-sectional study*—Give the eligibility criteria, and the sources and methods of selection of participants	5-6	**Methods -** Eligibility for participation in the surveys & Recruitment of participants to participate in the survey
		(*b*) *Cohort study*—For matched studies, give matching criteria and number of exposed and unexposed<br>*Case-control study*—For matched studies, give matching criteria and the number of controls per case		N/A
Variables	7	Clearly define all outcomes, exposures, predictors, potential confounders, and effect modifiers. Give diagnostic criteria, if applicable	9-12	**Methods –** Outcome Measures

Data sources/ measurement	8*	For each variable of interest, give sources of data and details of methods of assessment (measurement). Describe comparability of assessment methods if there is more than one group	9-12	**Methods –** Outcome Measures
Bias	9	Describe any efforts to address potential sources of bias	9-12	**Methods –** Outcome Measures
Study size	10	Explain how the study size was arrived at	13	**Methods** – Data Analysis

Continued on next page

Quantitative variables	11	Explain how quantitative variables were handled in the analyses. If applicable, describe which groupings were chosen and why	13	**Methods** – Data Analysis
Statistical methods	12	(*a*) Describe all statistical methods, including those used to control for confounding	12-13	**Methods** – Data Analysis
		(*b*) Describe any methods used to examine subgroups and interactions		N/A
		(*c*) Explain how missing data were addressed		**Methods** – Data Analysis
		(*d*) *Cohort study*—If applicable, explain how loss to follow-up was addressed<br>*Case-control study*—If applicable, explain how matching of cases and controls was addressed<br>*Cross-sectional study*—If applicable, describe analytical methods taking account of sampling strategy		N/A for sampling strategy
		(*e*) Describe any sensitivity analyses		N/A
**Results**				
Participants	13*	(a) Report numbers of individuals at each stage of study—eg numbers potentially eligible, examined for eligibility, confirmed eligible, included in the study, completing follow-up, and analysed	4, 13	**Methods - Figure 1.** Flow of participants through the study<br><br>**Results -** Characteristics of cross-sectional survey participants
		(b) Give reasons for non-participation at each stage	15-16	**Results** – Tables 2 & 3 (footnotes)
		(c) Consider use of a flow diagram		**Figure 1.** Flow of participants through the study
Descriptive data	14*	(a) Give characteristics of study participants (eg demographic, clinical, social) and information on exposures and potential confounders	13-14	**Results -** Characteristics of cross-sectional survey participants &<br><br>Table 1. Characteristics of birthing parents who participated in the 6- and 12-month survey, by HB4HNEKids participation
		(b) Indicate number of participants with missing data for each	14-15	**Results -** Table 1, 2 & 3 (footnotes)

		variable of interest		
		(c) *Cohort study*—Summarise follow-up time (eg, average and total amount)		N/A
Outcome data	15*	*Cohort study*—Report numbers of outcome events or summary measures over time		N/A
		Case-control study—Report numbers in each exposure category, or summary measures of exposure		N/A
		Cross-sectional study—Report numbers of outcome events or summary measures	*13-16*	**Results** – results have been reported separately for those exposed and not exposed to the HB4HNEKids program.
Main results	16	(*a*) Give unadjusted estimates and, if applicable, confounder-adjusted estimates and their precision (eg, 95% confidence interval). Make clear which confounders were adjusted for and why they were included	16-19	**Results -** Potential effectiveness<br>(All subheadings + Tables 4, 5 & 6)
		(*b*) Report category boundaries when continuous variables were categorized		N/A
		(*c*) If relevant, consider translating estimates of relative risk into absolute risk for a meaningful time period	16	**Results - Potential effectiveness**<br>Breastfeeding status, breastfeeding duration, introduction to solids and breastfeeding self-efficacy<br>"…at both time-points, HB4HNEKids participants reported a 5% higher point prevalence of breastfeeding compared with the comparison group participants."

Continued on next page

Other analyses	17	Report other analyses done—eg analyses of subgroups and interactions, and sensitivity analyses		N/A
**Discussion**				
Key results	18	Summarise key results with reference to study objectives	19	**Discussion** – Principal Results
Limitations	19	Discuss limitations of the study, taking into account sources of potential bias or imprecision. Discuss both direction and magnitude of any potential bias	21-22	**Discussion** – Limitations
Interpretation	20	Give a cautious overall interpretation of results considering objectives, limitations, multiplicity of analyses, results from similar studies, and other relevant evidence	19-21	**Discussion** – Comparison with Prior Work
Generalisability	21	Discuss the generalisability (external validity) of the study results	21	**Discussion** – Limitations<br>"…convenience sampling methods, which included only a small subsample of participants who had received the HB4HNEKids program, thus not providing data that is representative of the whole HNE region."
**Other information**				
Funding	22	Give the source of funding and the role of the funders for the present study and, if applicable, for the original study on which the present article is based	22	**Funding**

*Give information separately for cases and controls in case-control studies and, if applicable, for exposed and unexposed groups in cohort and cross-sectional studies.

Note: An Explanation and Elaboration article discusses each checklist item and gives methodological background and published examples of transparent reporting. The STROBE checklist is best used in conjunction with this article (freely available on the Web sites of PLoS Medicine at http://www.plosmedicine.org/, Annals of Internal Medicine at http://www.annals.org/, and Epidemiology at http://www.epidem.com/). Information on the STROBE Initiative is available at www.strobe-statement.org.
